# Age-Related Memory Impairment Is Associated with Increased zif268 Protein Accumulation and Decreased Rpt6 Phosphorylation

**DOI:** 10.3390/ijms21155352

**Published:** 2020-07-28

**Authors:** Sydney Trask, Brooke N. Dulka, Fred J. Helmstetter

**Affiliations:** Department of Psychology, The University of Wisconsin-Milwaukee, P.O. Box 413, Milwaukee, WI 53201, USA; trask@uwm.edu (S.T.); dulka@uwm.edu (B.N.D.)

**Keywords:** aging, immediate early gene, protein degradation, memory, proteasome

## Abstract

Aging is associated with cognitive decline, including impairments in the ability to accurately form and recall memories. Some behavioral and brain changes associated with aging are evident as early as middle age, making the understanding of associated neurobiological mechanisms essential to aid in efforts aimed at slowing cognitive decline throughout the lifespan. Here, we found that both 15-month-old and 22-month-old rats showed impaired memory recall following trace fear conditioning. This behavioral deficit was accompanied by increased zif268 protein accumulation relative to 3-month-old animals in the medial prefrontal cortex, the dorsal and ventral hippocampi, the anterior and posterior retrosplenial cortices, the lateral amygdala, and the ventrolateral periaqueductal gray. Elevated zif268 protein levels corresponded with decreases in phosphorylation of the Rpt6 proteasome regulatory subunit, which is indicative of decreased engagement of activity-driven protein degradation. Together, these results identify several brain regions differentially impacted by aging and suggest that the accumulation of proteins associated with memory retrieval, through reduced proteolytic activity, is associated with age-related impairments in memory retention.

## 1. Introduction

Normal human aging is accompanied by several changes in cognitive function [1], including impairments in memory formation and retention [2,3]. Age-related deficits have also been observed in the rodent literature, with aged animals demonstrating impairments in several memory paradigms, including the Morris water maze [4,5,6,7], the radial arm maze [8,9], active and passive avoidance learning [9,10,11], object recognition [12], and the objects in updated locations task [13]. Maintaining unimpaired cognitive ability in older adults is considered one of the major components to healthy aging [14], and understanding the neurobiological mechanisms that contribute to cognitive dysfunction is crucially important to facilitating healthy aging in a senescent human population [15].

Age-related memory deficits are also evident following trace fear conditioning (TFC), a type of classical conditioning in which the conditional stimulus (CS) and unconditional stimulus (UCS) are separated by a brief period of time known as a the “trace interval”. Older animals show reduced conditional responding (e.g., freezing) following CS–UCS pairings compared to young adult counterparts [16,17,18]. Interestingly, this age-related deficit is not seen in delay fear conditioning, in which a similar CS coterminates with the UCS [19]. This behavioral finding is believed to result from the age-related degeneration of a larger neural circuit required for the successful formation and recall of a trace, as opposed to delay, fear memory. The TFC circuit includes the hippocampus [20,21,22,23], retrosplenial cortex (RSC; [24,25,26]), and medial prefrontal cortex (mPFC; [27,28]) in addition to brain areas such as the lateral amygdala (LA) and the ventrolateral periaqueductal gray (vlPAG), which are also necessary for the delay version of the procedure [29,30]. Interestingly, the basolateral amygdala (BLA; [16]), dorsal hippocampus (DH; [16,31]), ventral hippocampus (VH; [31]), mPFC [6,16,32], and RSC [33] all show neurobiological changes in aged rodents relative to their young-adult counterparts. These changes range from impairments in protein degradation associated with memory retrieval [16] to changes in resting state neural activity [33]. While decreases in protein degradation processes occur as a function of normal aging [34], impairments in protein homeostasis and degradation are primary factors underlying age-associated protein accumulation [35,36]. Importantly, protein degradation following memory retrieval is necessary for the successful formation and stabilization of long-term memory [37,38].

The current experiment was designed to examine age-related brain changes throughout the trace fear circuit. We measured the degree of impaired trace fear recall as well as associated molecular changes throughout brain regions known to support TFC in young (3-month-old), middle-aged (15-month-old), and aged (22-month-old) rats. However, not all aged animals show deficits in TFC [17]. Several studies have demonstrated that memory impairments, and not age per se, correspond with molecular changes [5,16,39]. Aged animals that show no behavioral impairment often resemble young-adult counterparts on molecular endpoints. We predicted that deficits in memory retrieval would correspond with alterations in protein accumulation associated with memory retrieval. Specifically, we measured expression of the zinc finger transcription factor zif268 in several brain regions important for TFC (mPFC, anterior RSC, posterior RSC, DH, VH, BLA, and ventrolateral periaqueductal gray, or vlPAG). Zif268 is an immediate-early gene (IEG) that is necessary for the active process of memory retrieval and memory consolidation [40,41,42,43,44,45], and it also can serve as a general marker of increased neural activity [46,47,48]. Memory retrieval-related increases in zif268 are greater for aged animals than their young adult counterparts despite showing poorer recall, while baseline age differences are not typically seen in naïve controls [35,49]. Based on this, we predicted that age-related deficits in fear recall would be associated with increases in zif268 protein accumulation.

We were also interested in how changes in the ubiquitin proteasome system (UPS), which is critically needed for protein degradation and synaptic plasticity following memory activation, corresponded with differences in memory performance across the lifespan and how this related to the predicted elevation in zif268 protein expression. The UPS is a major regulatory pathway that is responsible for the recognition and clearance of unneeded, abnormal, or damaged proteins [50]. Work from our lab [38,51] and others [52] has previously shown that the UPS is also important for engaging reconsolidation-related synaptic mechanisms that are associated with memory retrieval, and the functionality of this system decreases with age [34,36]. Decreased UPS function has also been associated with increases in protein accumulation. For example [53], it has been demonstrated that increased protein levels of the IEG Activity Regulated Cytoskeleton Associated Protein (ARC) corresponded with decreased ubiquitin-mediated degradation. Increased phosphorylation of Rpt6 protein (i.e., pRpt6), a regulatory subunit of the proteasome important for protein degradation, is observed following learning [50,54], and age-related decreases in retrieval-induced Rpt6 phosphorylation have been associated with impaired performance in a trace fear conditioning paradigm [16]. Further, cognitive deficits in at least one mouse model of Alzheimer’s disease can be reversed by manipulations that increase the phosphorylation of the Rpt6 subunit [55]. We predicted that increases in the accumulation of zif268 protein in each region of interest (ROI) would be accompanied by a reduction in activity-driven phosphorylation of Rpt6, which is a proxy measure for activity-driven engagement of the UPS [50].

## 2. Results

### 2.1. Middle-Aged (15-Month-Old) and Old (22-Month-Old) Animals Show Behavioral Impairment during Trace Fear Retrieval Relative to Young (3-Month-Old) Animals

A 3 (Group: 3 mo, 15 mo, 22 mo) × Time Period (Pre-CS, CS–UCS, Post-CS) ANOVA was conducted to assess responding throughout the acquisition session (Means/SD were as follows. CS–UCS: 3 mo: 793.54/975.33; 15 mo: 156.36/66.52; 22 mo: 177.00/99.64. Post-CS: 3 mo: 831.54/1089.63, 15 mo: 137.56/63.65, 22 mo: 180.43/104.30). This found a main effect of time period, *F*_(2, 34)_ = 3.96, Mean Square Error (MSE) = 130796.71, *p* < 0.001, *η_p_*^2^ = 0.19, but no effect of group nor an interaction, largest *F* = 2.59, *p* = 0.11, indicating that all groups increased their freezing throughout the session in response to shock. Although the interaction was not significant, this effect appeared to be largest in the young animals, which may suggest that animals may acquire the learning differently as they age.

**Figure 1:**
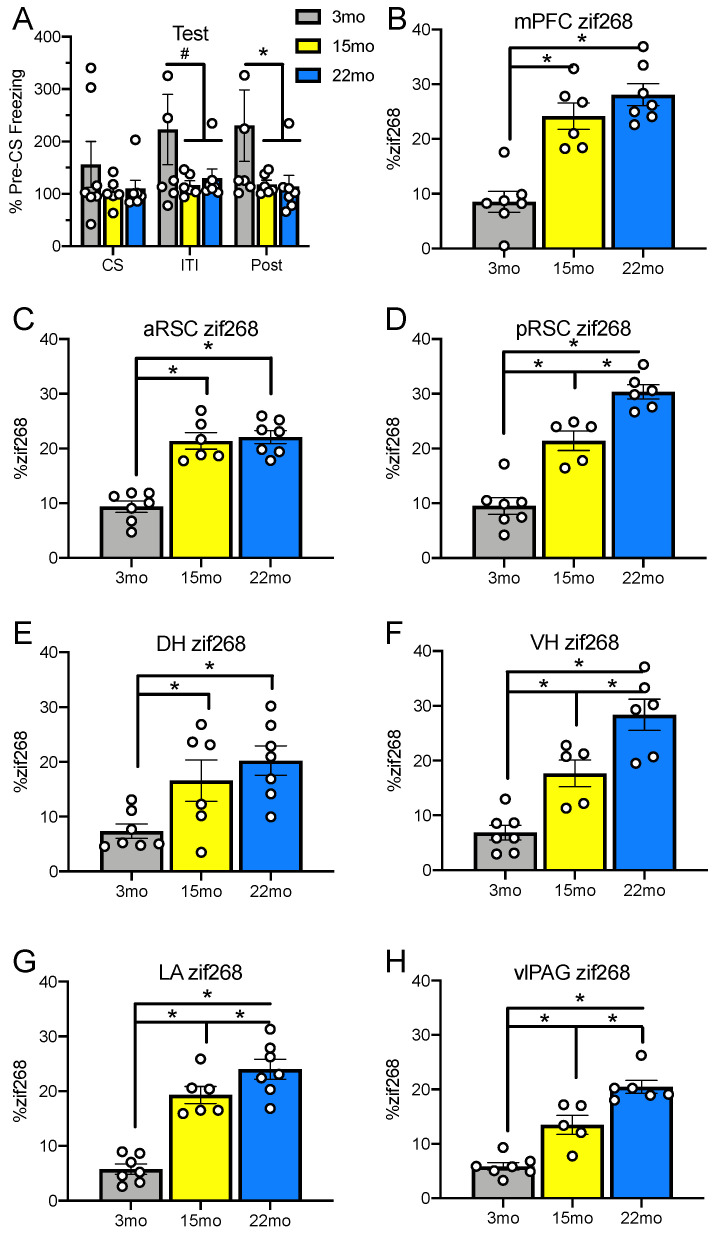
Behavioral results from the retrieval test in a neutral context (mean and SEM) are shown in Panel (**A**). Panels (**B**–**H**) show zif268 expression reported as a percentage (mean and SEM) of total DAPI present in the same section. Zif268 was increased in both 15-month-old and 22-month-old animals relative to 3-month-old animals in the medial prefrontal cortex (mPFC) (**B**), the aRSC (**C**), pRSC (**D**), dorsal hippocampus (DH) (**E**), ventral hippocampus (VH) (**F**), LA (**G**), and ventrolateral periaqueductal gray (vlPAG) (**H**). In the pRSC, VH, LA, and vlPAG, 22-month-old animals also showed increased zif268 relative to 15-month-old animals. Asterisks indicate *p* < 0.05; pound signs indicate *p* < 0.10. White dots represent individual rats.

Results from the memory retrieval test are depicted in Figure 1A. A 3 (Group: 3 mo, 15 mo, 22 mo) × Time Period (CS, ITI, Post-CS) ANOVA conducted to assess responding during this session found a main effect of time period, *F*_(2, 34)_ = 6.46, MSE = 1067.50, *p* = 0.004, *η_p_*^2^ = 0.28, a marginally significant interaction, *F*_(3, 34)_ = 2.57, MSE = 1067.50, *p* = 0.06, *η_p_*^2^ = 0.23, and no effect of group, *F* = 1.86, *p* = 0.19, despite a clear visual trend showing decreased freezing in middle-aged and old animals. Since group differences were not observed between 15-month-old and 22-month-old animals and animals have shown deficits in fear conditioning tasks as early as middle age [19], these groups were combined to increase statistical power and compared to young adult controls to provide a clearer follow up on the marginal interaction. This 2 (Group: 3 mo, 15 mo, and 22 mo) × Time Period (CS, ITI, Post-CS) ANOVA found a significant effect of time period, *F*_(2, 36)_ = 10.30, MSE = 1021.98, *p* < 0.001, *η_p_*^2^ = 0.36, an interaction, *F*_(2, 36)_ = 5.12, MSE = 1021.98, *p* = 0.01, *η_p_*^2^ = 0.22, and a marginal effect of group, *F*_(1, 18)_ = 3.94, MSE = 26570.66, *p* = 0.06. Planned comparisons showed that groups did not differ during the CS period, *p* = 0.16, showed a trend toward significance in the ITI, *p* = 0.06, and differed significantly during the Post-CS period, *p* = 0.04. Together, these results demonstrate that freezing was lower in the middle-aged and old animals compared to young animals.

### 2.2. The Amount of zif268 Protein Increases and the Phosphorylation of Rpt6 Decreases as a Function of Age in Brain Regions that Support Trace Fear Learning

**Figure 2:**
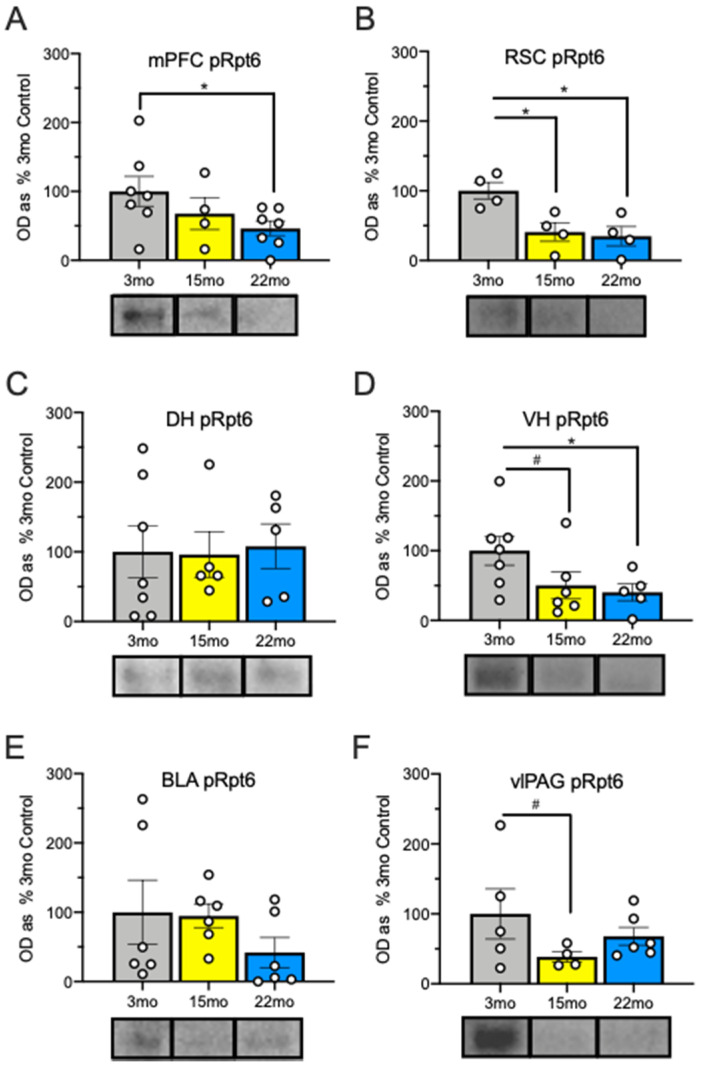
Rpt6 protein (pRpt6) reported as optical density as a percentage of 3-month-old control animals (mean and SEM) with representative images below group name. The 22-month-old animals showed decreased pRpt6 relative to the 3-month-old animals in the medial prefrontal cortex (mPFC) (**A**). In the retrosplenial cortex (RSC) (**B**), both the 15-month-old and 22-month-old animals showed decreased pRpt6. No differences were observed in the DH (**C**). The 22-month-old animals showed decreased pRpt6 in the VH (**D**), where a trend toward a decrease was seen in the 15-month-old animals. We found no differences in the basolateral amygdala (BLA) (**E**), and a trend toward a decrease in 15-month-old animals in the vlPAG (**F**). Asterisks indicate *p* < 0.05; pound signs indicate *p* < 0.10. White dots represent individual rats.

A one-way ANOVA comparing age group was conducted for each ROI for zif268 expression (Figure 1) as well as for changes in pRpt6 (Figure 2). Post-hoc Tukey’s Least Significant Difference (LSD) tests were conducted to examine group differences following the ANOVA. 

### 2.3. Medial Prefrontal Cortex

We first examined changes in zif268 accumulation and Rpt6 phosphorylation in the mPFC, which is a region that is important for working memory [56]. In line with this, the mPFC is needed to link related events separated by time in trace conditioning [28] and also shows age-related disruptions in UPS function [16]. Group differences in zif268 accumulation were found in the mPFC (Figure 1B), *F*_(2, 17)_ = 25.67, *p* < 0.001. Post-hoc tests showed that while 3-month-old animals were lower than 15-month-old (*p* < 0.001) and 22-month-old (*p* < 0.001) animals, the 15- and 22-month-old groups did not differ from each other (*p* = 0.21). While the ANOVA examining pRpt6 in the mPFC (Figure 2A), *F*_(2, 15)_ = 2.44, *p* = 0.12, was not significant, 22-month-old animals showed decreases in pRpt6 relative to 3-month-old animals, *p* = 0.04. No other between-group differences were found; the smallest *p* = 0.28. No group differences were found in total Rpt6, *F* < 1, or in actin, *F* = 1.06, *p* = 0.32, suggesting that the changes may be specific to proteins linked to memory-related neural activity. 

### 2.4. Retrosplenial Cortex

We next examined age-related changes in the RSC, which is important in the retrieval of trace, but not delay, fear conditioning [24,25] as well as spatial and contextual processing more generally [57]. Group differences were found in zif268 accumulation in the anterior RSC (aRSC; Figure 1C), *F*_(2, 17)_ = 34.93, *p* < 0.001. Post-hoc tests again showed that while accumulation in the 3-month-old animals was lower than that in both the 15-month-old (*p* < 0.001) and 22-month-old (*p* < 0.001) animals, these two groups did not differ from each other (*p* = 0.69). The ANOVA conducted to assess zif268 accumulation in the posterior RSC (pRSC) also was significant (Figure 1D), *F*_(2, 15)_ = 50.61, *p* < 0.001. Post-hoc tests revealed that all groups differed significantly from each other (3 mo vs. 15 mo: *p* < 0.001; 3 mo vs. 22 mo: *p* < 0.001; 15 mo vs. 22 mo: *p* = 0.001), with increases in expression during aging. When examining pRpt6 in fractionated samples that included tissue from both the anterior and posterior RSC (Figure 2B), the ANOVA was significant, *F*_(2, 9)_ = 7.63, MSE = 680.79, *p* = 0.01. Both 15-month-old, *p* = 0.01, and 22-month old animals, *p* = 0.006, showed reduced pRpt6 relative to 3-month-old animals but did not differ from each other, *p* = 0.75. There were no differences in total Rpt6 subunit protein or actin, *F*s < 1.

### 2.5. Dorsal Hippocampus

In the DH (Figure 1E), a region recruited for trace fear conditioning [21] and spatial learning [58], the ANOVA examining zif268 accumulation was again significant, *F*_(2, 17)_ = 6.51, *p* = 0.008. As in the mPFC and the aRSC, post-hoc tests demonstrated that while 3-month-old animals differed from both 15-month-old (*p* = 0.03) and 22-month-old (*p* = 0.003) animals, these two groups did not differ from each other (*p* = 0.35). No systematic changes in pRpt6 were seen in the DH (Figure 2C), *F* < 1, and no group differences were observed, smallest *p* = 0.83. The same was true of total Rpt6, *F* < 1, and of actin, *F* = 2.83, *p* = 0.12.

### 2.6. Ventral Hippocampus

Then, we examined zif268 accumulation and pRpt6 in the ventral hippocampus (VH), which is a region needed for both the acquisition and expression of trace fear [23] as well as spatial processing [58]. Groups also differed in zif268 accumulation in the VH (Figure 1F), *F*_(2, 15)_ = 25.74, *p* <0.001, with post-hoc tests revealing that all groups differed from each other (3 mo vs. 15 mo: *p* = 0.004; 3 mo vs. 22 mo: *p* < 0.001; 15 mo vs. 22 mo: *p* = 0.005). When examining pRpt6 in the VH (Figure 2D), there was a marginal interaction, *F*_(2, 15)_ = 2.98, MSE = 2153.87, *p* = 0.08. The 22-month-old animals showed less pRpt6 than the 3-month-old animals, *p* = 0.04, and there was a marginal decrease between the 3- and 15-month-old animals, *p* = 0.07, while the 15-month-old and 22-month-old animals did not differ from each other, *p* = 0.73. No differences were observed in total Rpt6 or actin, *F*s < 1.

### 2.7. Lateral Amygdala

In the LA (Figure 1G), a region important for both trace and delay fear conditioning [59,60], the ANOVA to test for differences in zif268 accumulation was significant, *F*_(2, 17)_ = 41.99, *p* < 0.001. Post-hoc tests revealed that all groups again differed from each other (3 mo vs. 15 mo: *p* < 0.001; 3 mo vs. 22 mo: *p* < 0.001; 15 mo vs. 22 mo: *p* = 0.04). When examining pRpt6 in tissue that included the entire basolateral amygdala complex (Figure 2E), the ANOVA was not significant, *F*_(2,15)_ = 1.07, *p* = 0.37, and group differences were not observed, smallest *p* = 0.21. There were also no differences in total Rpt6 or actin, *F*s < 1.

### 2.8. Ventrolateral Periaqueductal Gray

Finally, we examined zif268 accumulation and Rpt6 phosphorylation in the ventrolateral periaqueductal gray (vlPAG), which is a region that is important for timing and generation of the fear response [61] as well as error correction during memory acquisition [30]. Groups differed in zif268 accumulation in the vlPAG (Figure 1H), *F*_(2, 15)_ = 41.07, *p* < 0.001. Post-hoc tests showed that all groups differed from each other (3 mo vs. 15 mo: *p* < 0.001; 3 mo vs. 22 mo: *p* < 0.001; 15 mo vs. 22 mo: *p* = 0.001), in a pattern similar to the pRSC, VH, and LA. The ANOVA testing for differences in pRpt6 was not significant in the vlPAG (Figure 2F), *F*_(2, 12)_ = 1.62, *p* = 0.24, with a trend in differences only between the 3-month-old and 15-month-old animals, *p* =0.10, but no others, smallest *p* = 0.32. No differences were observed in total Rpt6, *F* = 1.25, *p* =0.32, or actin, *F* = 1.46, *p* = 0.24, indicating once again that protein accumulation was specific to proteins actively engaged by memory retrieval.

Together, these results show that aging results in an increase in expression of the zif268 protein throughout the trace fear circuit, and several of these regions have corresponding decreases in the phosphorylation of the Rpt6 proteasome regulatory subunit.

### 2.9. Age-Related Changes in zif268 and pRpt6 are Associated with Degree of Behavioral Impairment

**Figure 3:**
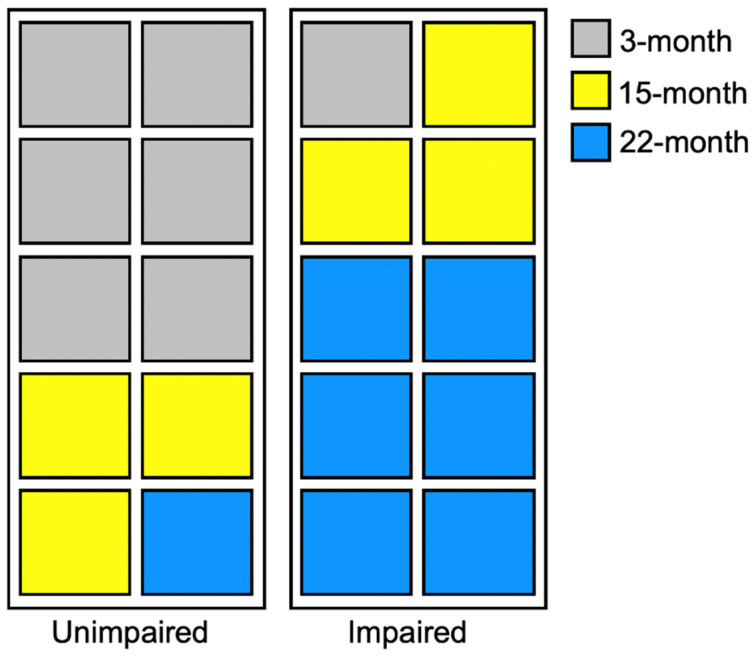
Schematic showing animals from each group classified as unimpaired or impaired based on behavioral performance. Each square represents an individual animal.

**Figure 4:**
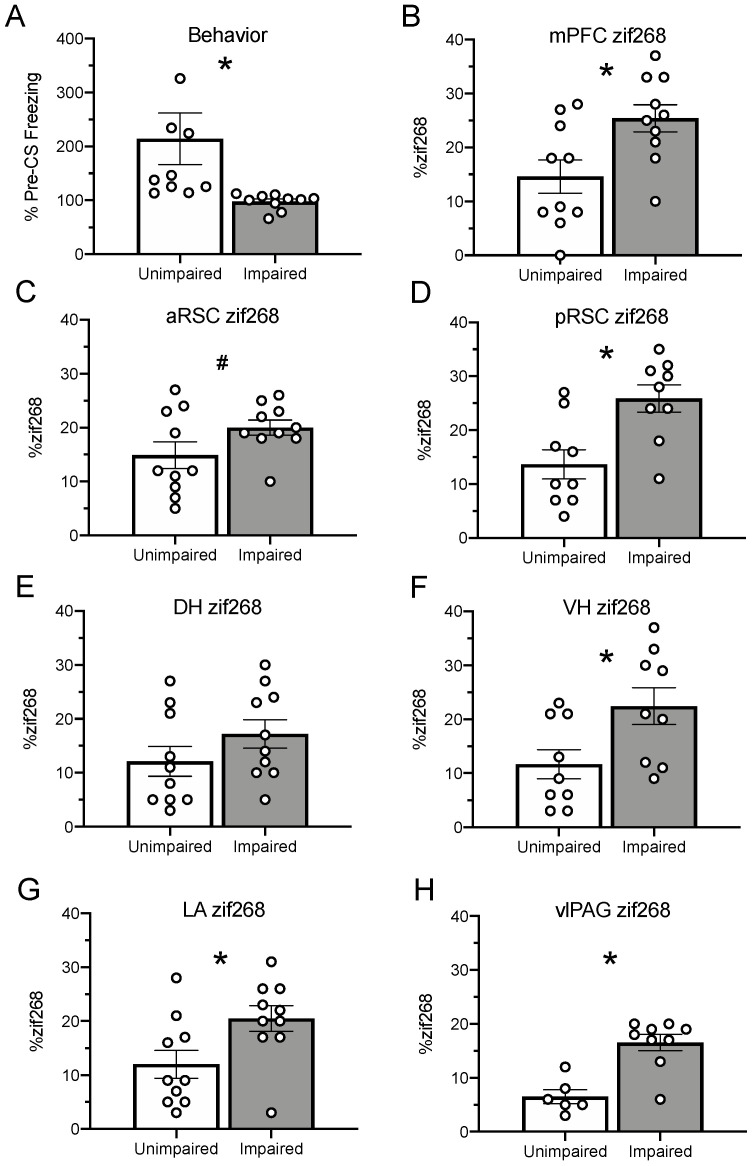
Animals were divided into two groups (high freezers and low freezers) based on a median split of performance in the Post-CS (conditional stimulus) period during the retrieval test. Results from the Post-CS period during the retrieval test (mean and SEM) are shown in Panel (**A**), where impaired animals froze less than unimpaired animals. Panels (**B**–**H**) show zif268 expression reported as a percentage (mean and SEM) of total DAPI present in the same section. Impaired animals had increased zif268 relative to unimpaired in the mPFC (**B**). While there was a trend in the aRSC (**C**), impaired animals showed increased zif268 in the pRSC (**D**). No differences were seen in the DH (**E**), but impaired animals had increased zif268 relative to unimpaired animals in the VH (**F**). This finding held in the LA (**G**) and the vlPAG (**H**). Asterisks indicate *p* < 0.05; pound signs indicate *p* < 0.10. White dots represent individual rats.

Based on the above findings, we hypothesized that decreased memory retention, as indicated by reduced freezing, would be associated with increased zif268 expression. To test this, animals were split into two groups based on a median split of their Post-CS freezing during the test [16,39]. The high freezers (Group Unimpaired; *n* = 10) consisted of six 3-month-old, three 15-month-old, and one 22-month-old animal. The low freezers (Group Impaired; *n* = 10) were comprised of one 3-month-old, three 15-month-old, and six 22-month-old animals. A schematic representing group membership after the median split was applied as depicted in Figure 3. All data were analyzed using independent samples t-tests. As defined by a median split on post-CS freezing behavior, impaired animals froze less than the unimpaired (Figure 4A), *t*_(18)_ = 2.43, *p* = 0.03. Impaired animals showed greater zif268 expression than unimpaired animals in the mPFC (Figure 4B; *t*_(18)_ = 2.74, *p* = 0.01), pRSC (Figure 4C; *t*_(16)_ = 2.32, *p* = 0.005), VH (Figure 4F; *t*_(16)_ = 2.47, *p* = 0.03), LA (Figure 4G; *t*_(18)_ = 2.52, *p* = 0.02), and vlPAG (Figure 4H; *t*_(16)_ = 2.75, *p* = 0.01). While this visual trend appeared to hold on the aRSC (Figure 4D; *t*_(18)_ = 1.75, *p* = 0.10) and the DH (Figure 4C; *t*_(18)_ = 1.32, *p* = 0.20), neither reached significance. 

**Figure 5:**
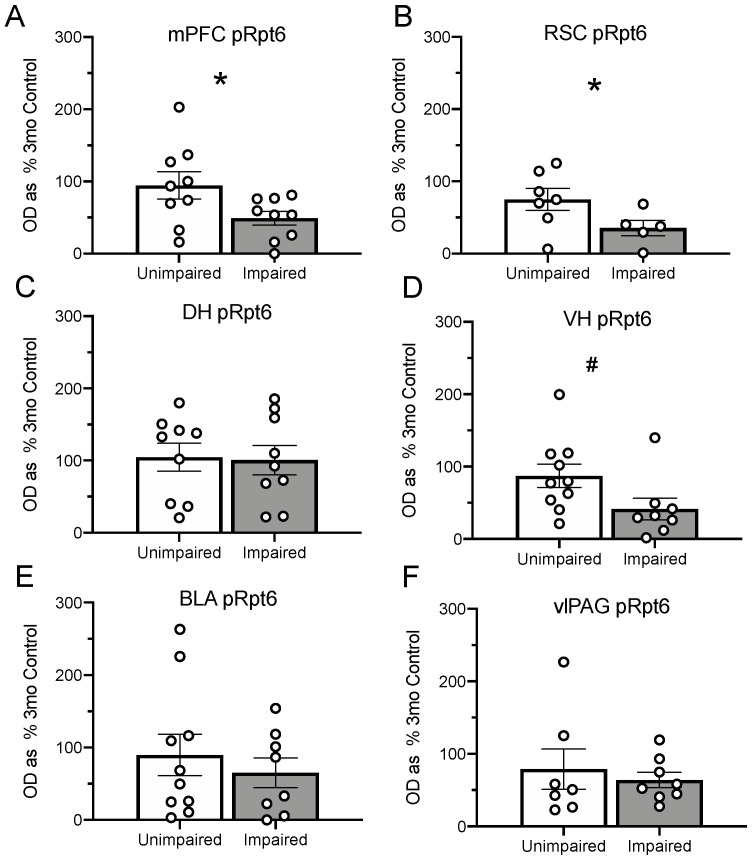
Animals were divided into two groups based on a median split of performance in the Post-CS period during the retrieval test. High freezers were designated as unimpaired, whereas low freezers were designated as impaired. Panels (**A**–**F**) show pRpt6 reported as a percentage (mean, SEM) in 3-month-old animals. Impaired animals showed less pRpt6 than unimpaired in both the mPFC (**A**) and the RSC (**B**). As before, there were no differences in the DH (**C**). Impaired memory performance corresponded with a trend toward a decrease in the VH (**D**). No differences between unimpaired and impaired animals were observed in the LA (**E**) or the vlPAG (**F**). Asterisks indicate *p* < 0.05; pound signs indicate *p* < 0.10. White dots represent individual rats.

When examining the data as a function of behavioral performance using the median split procedure describe above, impaired animals showed decreases in pRpt6 in the mPFC (Figure 5A, *t*_(16)_ = 2.15, *p* = 0.047) as compared to unimpaired animals. There was a trend in the RSC (Figure 5B, *t*_(10)_ = 1.96, *p* = 0.08) and the VH (Figure 5D, *t*_(16)_ = 2.04, *p* = 0.06), but this was not the case in the DH, (Figure 5C, *t*_(15)_ = 0.99, *p* = 0.34), LA (Figure 5E, *t*_(16)_ = 0.66, *p* = 0.52), and vlPAG (Figure 5F, *t*_(13)_ = 0.54, *p* = 0.60).

In order to test if memory impairment was correlated with zif268 expression, post-CS behavior during retrieval was transformed using a Log10 transformation (to compress extreme scores) and Pearson’s bivariate correlations were run with each region of interest. Zif268 expression was negatively correlated with behavior in the mPFC (*r* = −0.38, *p* = 0.05), pRSC (*r* = −0.50, *p* = 0.02), VH (*r* = −0.50, *p* = 0.02), and LA (*r* = −0.37, *p* = 0.05), demonstrating that decreases in performance corresponded with increased protein. While this trend appeared in the aRSC (*r* = −0.33, *p* = 0.08), DH (*r* = −0.24, *p* = 0.15), and vlPAG (*r* = −0.31, *p* = 0.11), it was not significant. Similar correlations examined the relationship between pRpt6 and behavior. Rpt6 phosphorylation was positively correlated with behavior in the RSC (*r* = 0.68, *p* = 0.008) and the VH (*r* = 0.45, *p* = 0.03), demonstrating that reductions in pRpt6 were associated with impaired behavioral performance. This was not the case in the mPFC (*r* = 0.27, *p* = 0.14), DH (*r* = −0.27, *p* = 0.11), LA (*r* = −0.06, *p* = 0.40), or vlPAG (*r* = 0.02, *p* = 0.47). 

### 2.10. Decreased pRpt6 Corresponds with Increased Levels of zif268 in Several Brain Regions

**Figure 6:**
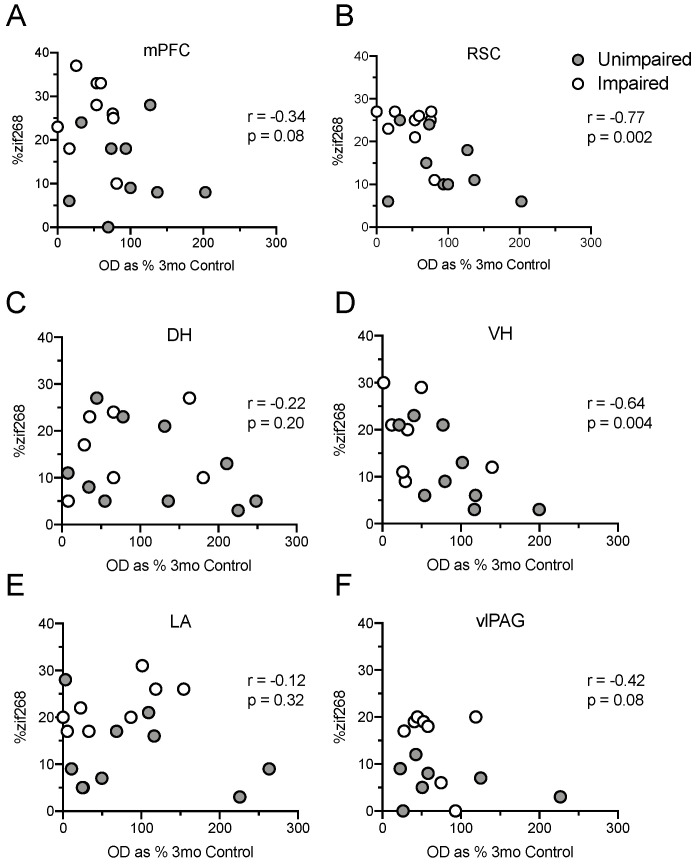
Scatterplots depicting correlations between phosphorylated Rpt6 and zif268 accumulation in the mPFC (**A**), RSC (**B**), DH (**C**), VH (**D**), LA (**E**), and vlPAG (**F**). Unimpaired animals are represented by gray dots, and impaired animals are represented by white dots.

In each region of interest, we next tested if Rpt6 phosphorylation was associated with increases in zif268 accumulation (Figure 6). This was the case in the RSC (Figure 6B; *r* = −0.77, *p* = 0.002) and the VH (Figure 6D; *r* = −0.64, *p* = 0.004), with a trend toward this association in the mPFC (Figure 6A; *r* = −0.34, *p* = 0.08) and the vlPAG (Figure 6F; *r* = −0.42, *p* = 0.08). Decreases in pRpt6 were not associated with zif268 accumulation in the DH (Figure 6C; *r* = −0.22, *p* = 0.20) or the LA (Figure 6E; *r* = −0.12, *p* = 0.32).

### 2.11. Age-Related zif268 and pRpt6 Changes Hold throughout the Trace Fear Circuit

**Figure 7:**
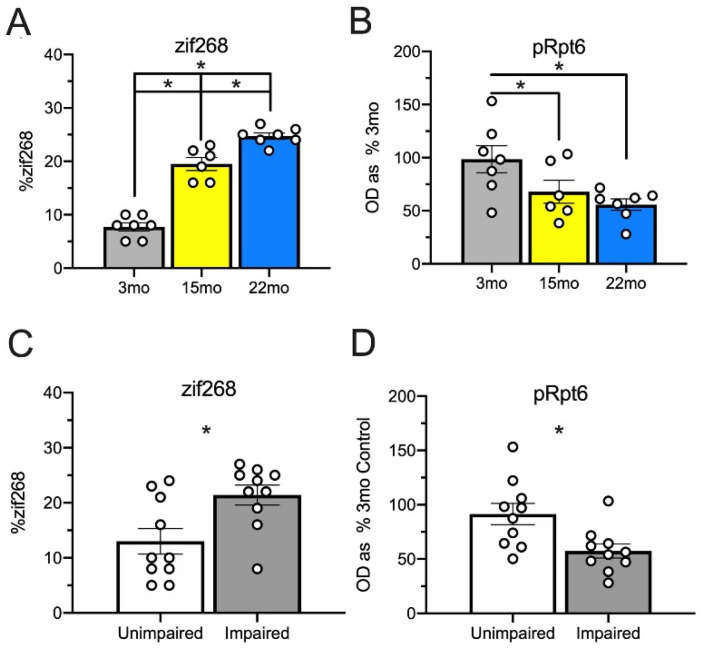
Levels of zif268 expression and pRpt6 in each ROI were averaged throughout the entire brain for each animal. Panels (**A**,**B**) represent zif268 (**A**) and pRpt6 (**B**) levels with groups constructed based on age group. zif268 levels differ between all age groups (**A**). Both 15-month-old and 22-month-old animals showed less pRpt6 than 3-month-old animals, but did not differ from each other. (**B**) Panels (**C**,**D**) represent zif268 (**C**) and pRpt6 (**D**) levels with groups constructed based on behavioral performance defined by a median split. Animals with impaired memory performance showed increased zif268 (**C**) and decreased pRpt6 (**D**) relative to unimpaired animals. Asterisks indicate *p* < 0.05. White dots represent individual rats.

Each animal’s levels of zif268 expression and pRpt6 within each ROI and subject were examined in the current study and averaged. An ANOVA examining zif268 expression throughout the brain was significant (Figure 7A), *F*_(2, 17)_ = 104.02, MSE = 5.08, *p* < 0.001. All groups differed from each other (3 mo vs. 15 mo: *p* < 0.001; 3 mo vs. 22 mo: *p* < 0.001; 15 mo vs. 22 mo: *p* = 0.001). A similar ANOVA assessing pRpt6 levels (Figure 7B) throughout the whole brain was also significant, *F*_(2, 17)_ = 4.95, MSE = 681.02, *p* = 0.02. Both 15-month-old (*p* = 0.05) and 22-month-old animals (*p* = 0.007) showed less pRpt6 than the 3-month controls but did not differ from each other (*p* = 0.42), demonstrating that Rpt6 phosphorylation is reduced with age.

Then, we examined whether global changes throughout the brain were associated with behavior using the median split analysis described above. Impaired memory performance corresponded with increased zif268 accumulation (Figure 7C), *t*_(18)_ = 2.84, *p* = 0.01, and decreased pRpt6 (Figure 7D), *t*_(18)_ = 2.88, *p* = 0.10, relative to unimpaired animals. Finally, we examined how 22-month-old animals differed from young and middle-aged animals as a group on these measures. The 22-month-old animals showed increased zif268, *t*_(18)_ = 4.52, *p* < 0.001, and decreased pRpt6, *t*_(18)_ = 2.14, *p* = 0.046.

## 3. Discussion

The present findings demonstrate age-related changes in zif268 accumulation and Rpt6 phosphorylation throughout the brain. Aging resulted in increases in zif268 protein content, with both the 15-month and 22-month groups showing increased zif268 accumulation relative to the 3-month animals in all selected ROIs. However, increased zif268 accumulation in the 22-month-old animals relative to the 15-month-old animals was evident in several regions. In the posterior RSC, VH, LA, and the vlPAG, 22-month-old animals showed elevated zif268 expression relative to the 15-month-old animals in addition to the 3-month-old animals. This was not the case in the mPFC, aRSC, and the DH. Interestingly, all regions of interest showed an association between freezing during the test and zif268 expression where lower levels of freezing were associated with higher levels of zif268 expression, except for the DH [6] and the aRSC. These results are also consistent with hypotheses that suggest age-related memory impairments result from an increase in IEG accumulation as a result of reduced proteolytic activity and subsequent protein degradation rather than an overall decrease in number of neurons [35,62]. These age-related decreases in zif268 degradation were largely accompanied by decreases in pRpt6. The clearest effects were observed in regions known to be critical for trace conditioning, such as the mPFC, RSC, and VH. Interestingly, the age-related differences we observed in several brain regions following trace fear retrieval were specific to proteins associated with memory processing (zif268 and pRpt6). Total levels of Rpt6 and actin, proteins that are typically not affected by memory retrieval, were unaffected. Together, these results suggest that the IEG-clearing process following memory retrieval, rather than global protein accumulation, is reduced in older animals. Thus, cognitive impairments might instead arise from decreases in activity-driven protein degradation rather than protein accumulation itself. 

The current results support prior work demonstrating that memory impairments in aged animals are associated with increases in retrieval-induced zif268 protein expression [49], as well as other IEGs [63] and provide a likely mechanism through which protein accumulation occurs. These results are consistent with others that have found indications of increased neural activity in older animals that show memory deficits. For example, age-related deficits in the Morris water maze corresponded with increased mRNA levels in the hippocampus [64,65]. One interesting discrepancy in this literature is that following TFC learning, zif268 expression in aged mice is reduced relative to young adult controls [66]. While the current results might be at odds with this finding, they also may suggest that the pattern of zif268 activity differs between initial memory consolidation and later memory retrieval, although to our knowledge, this has yet to be systematically tested. 

These results are the first to show memory-related protein accumulation and UPS engagement associated in brain regions known to support trace fear across three different age groups. The results from the 15-month age group are especially interesting because of the intermediate nature of both their behavioral performance and their biological outcomes. Recall that while overall middle-aged animals seemed to resemble old animals, an equal number of 15-month animals were classified as unimpaired or impaired when groups were constructed on a median split of memory performance. Furthermore, when examining zif268 accumulation in several brain regions, 15-month-old animals were more likely to be in an intermediate zone between the 3-month-old and 22-month-old animals. As is the case in the current study, the inclusion of a “middle-aged” group in studies on age-related cognitive decline often suggests that impairments develop over time and might exist on a spectrum. Recent findings from our own laboratory have demonstrated that using a stronger TFC protocol than the one employed in the current experiment (i.e., 10 CS–UCS pairings instead of 6), the 15-month-old animals demonstrated a less robust behavioral impairment than the 22-month-old animals [16]. Together, these results suggest that there are important boundary conditions on behavioral impairments and their associated cellular changes that are relatively understudied in middle-aged groups. Understanding the conditions that produce deficits in middle-aged cohorts may allow for more targeted preventative treatments before age-related decline becomes pathological.

One interesting dissociation observed in the present experiment is that while the 15-month-old and 22-month-old animals did not differ in zif268 expression in the anterior portion of the RSC, they did differ in zif268 expression in the posterior region. Other work in our lab has demonstrated dissociable roles for these regions in the acquisition of TFC in that the aRSC seems to be important for acquiring CS-related information, whereas the pRSC seems to be important for acquiring context-related information [67]. In the current experiment, the behavioral impairment associated with learning about the CS (as all testing occurred in a novel context) corresponded with activity in the aRSC in both 15-month-old and 22-month-old animals, but the 22-month-old animals showed greater activity than the 15-month-old animals in the pRSC. This suggests that if tested for conditional responding to acquisition context, 22-month-old animals might show an even more robust behavioral impairment that 15-month-old animals. A similar dissociation was demonstrated in zif268 and pRpt6 expression between the DH and VH, which is in line with other experiments that have demonstrated different functions of these regions in TFC [23,68]. 

Some clear limitations exist in the present study. Since we did not include a no-retrieval control condition, it is impossible to determine if the increases in zif268 accumulation observed were pre-existing or instead a specific result of memory retrieval. One explanation of the current data is that retrieval results in zif268 protein expression that is equivalent between groups, but failures in protein degradation in middle-aged and aged animals result in greater protein accumulation in the latter groups. A second explanation could be that aged brains create more IEG protein in general, and this process is independent of retrieval. However, recall that retrieval resulted in increases in zif268 protein based on age group, which is an effect that is not observed in naïve animals [49]. This suggests that the effect observed here is more likely driven by active memory retrieval processes than baseline differences in protein expression. Furthermore, the effects in the present manuscript were exclusive to proteins associated with memory retrieval (zif268 and pRpt6), as actin and total Rpt6 did not differ as a function of age in any region. 

Additionally, brain regions that have not been shown to be important in trace fear conditioning were not examined in the present study. Thus, it is impossible to tell if the pattern observed in the current dataset is unique to regions that are involved in TFC, or if this pattern holds throughout the entire brain even in regions not typically associated with the trace conditioning task. However, it should be noted that the strongest effects in the present experiment were in regions that are preferentially recruited during TFC and were weaker in regions that are recruited in both TFC and DFC, a task in which aged animals show no deficits [19].

Finally, given that older animals show increases in baseline freezing relative to young animals in both of our sampled time points (acquisition and retrieval), we must acknowledge that freezing is an inherently confounded measure when analyzing memory across age groups, and therefore, the behavioral conclusions must be approached with appropriate caution as increases in nonassociative freezing might result in a ceiling effect that leaves us unable to detect group differences. While we aimed to eliminate this confound using a within-subject correction to account for differences in pre-CS freezing, baseline freezing and CS-elicited freezing measures do often interact [69]. Therefore, the present behavioral data should be taken in concert with the corresponding descriptive molecular data, as well as understood within the greater context in cognitive decline overall as a function of age. Regardless of whether or not the observed behavioral effects were due to a ceiling effect, behavioral performance using the baseline-corrected metric, and not necessarily age, was tightly associated with zif268 accumulation and pRpt6 throughout the brain (see Figure 7C,D). 

## 4. Methods

### 4.1. Subjects

Subjects were 20 male Fisher 344 (F344) rats obtained from the National Institute on Aging (Charles River; Raleigh, NC, USA) at the ages of 3 (*n* = 7), 15 (*n* = 6), and 22 months (*n* = 7) old at the time of delivery. Animals were housed individually in plastic cages with chip bedding and free access to food in water. The room where animals were housed was maintained on a 14:10 light/dark cycle. All animals were run with approval from the University of Wisconsin-Milwaukee Institutional Animal Care and Use Committee (Protocol 2019-31, Approved 30 April 2019) in an AAALAC-accredited facility.

### 4.2. Behavioral Procedure

For TFC, animals were placed in a Med Associates (St. Albans, VT, USA) conditioning chamber (30.5 × 24.1 × 29.2 cm) housed in individual sound attenuating chambers. Chambers were illuminated with an incandescent house light, and exhaust fans provided a 65-dB background noise. A scent was created by cleaning each chamber with a 5% acetic acid solution immediately before the animal was placed in the chamber. Following a six-minute baseline period, rats received six CS–UCS pairings. The CS was a 10-s 72 dB white noise stimulus played from a speaker mounted to the wall of the sound-attenuating chamber. The UCS was a 1 mA footshock. A 20-s trace interval period separated each CS and UCS and the intertrial interval (ITI) between these pairings was on average 240 s. Animals remained in the chamber for four minutes following the final footshock. 

Animals were tested in a novel context (20.5 × 26.5 × 21 cm) for conditional freezing to the white noise CS. These conditioning boxes were housed in a sound-attenuating chamber in a separate room in the laboratory. The chamber was not illuminated, and the conditioning box had plexiglass flooring. To create a scent, it was cleaned with a 100% ethanol solution immediately before the animals were placed inside. Following a four-minute baseline period, animals received four 30-s CS presentations (with an average ITI of 60 s). Animals remained in the chamber for one minute following the final CS presentation. 

Freezing was defined as the cessation of all movement excluding respiration and was automatically scored in real time with FreezeScan 1.0 detection software (Clever Sys, Inc., Reston, VA, USA) calibrated to a trained human observer. To account for differences observed in baseline freezing to the chambers in older animals (Acquisition Context (mean/SD): 3 mo: 22.59/19.75; 15 mo: 53.88/30.45; 22 mo: 40.86/21.38. Testing Context: 3 mo: 32.14/35.56; 15 mo: 61.96/29.36; 22 mo: 65.26/24.31), results are shown as a percentage of each animal’s pre-CS responding throughout the test session [70]. This minimizes potential variation in behavior produced by factors unrelated to associative learning and instead captures the amount of behavior change produced by recall of the CS–UCS learning.

### 4.3. Immunofluorescence (IF)

**Figure 8:**
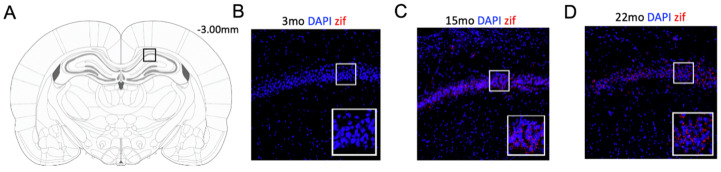
Schematic of coronal section where dorsal hippocampus (DH) slices were taken at −3.00 mm relative to bregma (Panel **A**). Representative images from taken at 20× 3-month-old (Panel **B**), 15-month-old (Panel **C**), and 22-month-old (Panel **D**) animals have DAPI depicted in blue and zif268 depicted in red. Insets of the highlighted region are shown in the bottom right corner for each image.

Animals were deeply anesthetized with isoflurane 60 min following the retrieval session. Previous research in our lab has demonstrated that zif268 expression peaks at 60 min following fear learning [71]. Brains were immediately removed and stored at −80 °C until sliced in 20-micron sections and mounted onto charged slides. Slides were rehydrated in wash buffer (PBS + 0.05% Tween-20) and permeabilized (PBS + 0.3% Triton X) for 15 min and incubated in blocking solution (PBS + 0.7% NGS). Then, slides were incubated in zif268/Erg-1 antibody (Cell Signaling, 1:500, #4153, Beverly, MA, USA) solution (PBS + 0.3% Triton X + 5% NGS) overnight at 4 °C. The next day, slides were incubated in secondary antibody solution for 2 h and rinsed with wash buffer, a DAPI counterstain was applied, and slides were cover slipped. Images were captured on an Olympus Fluoview FV1200 (Olympus, Center Valley, PA, USA) confocal microscope using a 20× objective lens. Serial z-stack images covered a depth of 4.55 μm through five consecutive sections (0.91 μm per section) and were acquired using Olympus Fluoview software. Zif268 activity was normalized as a proportion of DAPI present in the same section. The total amount DAPI did not differ between groups in any ROI, *F*s < 1, indicating that the overall amount of cells present did not depend on age. Representative images from the DH are depicted in Figure 8.

### 4.4. Crude Synaptosomal Membrane Fractionation

Synaptosomal membrane fractions were obtained using methods previously described [38,46] with minor alterations noted below. Tissue samples were homogenized in TEVP buffer with 320 mM Sucrose and centrifuged at 1000× *g* for 10 min at 4 °C. The supernatant was collected and spun at 10,000× *g* for 10 min at 4 °C. The resulting pellet containing the synaptosomal fraction was resuspended in phospho-homogenization buffer (50 mM Tris-HCl, 6 mM sodium deoxycholate, 150 mM NaCl, 1 mM NaF, two mini EDTA-free complete protease inhibitor tablets, 0.1% SDS, 1 mM sodium orthovanadate) and measured using a 660 nm protein assay (Pierce, Rockford, IL, USA). 

### 4.5. Western Blotting

Following synaptosomal preparation, protein levels were normalized and loaded onto a 7.5% SDS/PAGE gel and then to a membrane using a transfer apparatus (Bio-Rad, Richmond, CA, USA). Membranes were incubated in blocking buffer for 1 h before being incubated in pRpt6 (ProSci, 1:850, Poway, CA, USA), total Rpt6 (Abcam, 1:1000, Cambridge, MA, USA), or βactin (Cell Signaling, 1:1000, Beverly, MA, USA) primary solutions overnight at 4 °C. Then, membranes were incubated in the appropriate secondary (Cell Signaling, 1:20,000) antibody for either four hours at 4 °C (pRpt6 and tRpt6) or one hour at room temperature (βactin) and prepped in a chemiluminescence solution for 3 min. Images were captured and densitometry was performed using NIH Genesys. The pRpt6 antibody was generated commercially (ProSci) against a synthetic peptide (NH2-CALRND(pS)YTLHK-OH) as described previously [16]. Western blotting was conducted in the same regions as IF images were taken except as follows. Tissue punches from both the anterior and posterior RSC were fractionated together into one sample and from the entire basolateral amygdala complex rather than just the lateral amygdala. Then, 15 μg of protein was loaded into each well per animal per ROI for blotting.

### 4.6. Statistical Analysis

All results were analyzed using analyses of variance (ANOVAs) with planned comparisons to examine between-group differences following repeated measures ANOVAs and Tukey’s LSD to examine between-group differences following one-way ANOVAs, t-tests, or one-tailed Pearson’s bivariate correlation using SPSS (Statistical Package for Social Sciences, Version 25; IBM) software, with alpha set to *p* < 0.05. 

## 5. Conclusions

In conclusion, these findings increase our understanding of the molecular mechanisms associated with age-related cognitive decline by examining IEG activity throughout the trace fear circuit across an age spectrum in rats. While these results suggest that some brain regions (i.e., mPFC, aRSC, and DH) stop showing age-related changes at earlier ages, others (i.e., pRSC, VH, LA, and vlPAG) continue to change from middle age to senescent populations. Furthermore, they suggest that one likely mechanism through which increased protein expression might occur is through decreases in activity-driven protein degradation processes and subsequent protein accumulation.
